# MR Imaging of Prostate Cancer: Diffusion Weighted Imaging and (3D) Hydrogen 1 (^1^H) MR Spectroscopy in Comparison with Histology

**DOI:** 10.1155/2011/616852

**Published:** 2010-07-20

**Authors:** J. Yamamura, G. Salomon, R. Buchert, A. Hohenstein, J. Graessner, H. Huland, M. Graefen, G. Adam, U. Wedegaetner

**Affiliations:** ^1^Department of Diagnostic and Interventional Radiology, University Medical Center Hamburg-Eppendorf, 20246 Hamburg, Germany; ^2^Department of Urology, University Medical Center Hamburg-Eppendorf, 20246 Hamburg, Germany; ^3^Siemens AG, 20099 Hamburg, Germany

## Abstract

*Purpose.* To evaluate retrospectively the impact of diffusion weighted imaging (DWI) and (3D) hydrogen 1 (^1^H) MR-spectroscopy (MRS) on the detection of prostatic cancer in comparison to histological examinations. *Materials and Methods:* 50 patients with suspicion of prostate cancer underwent a MRI examination at a 1.5T scanner. The prostate was divided into sextants. Regions of interest were placed in each sextant to evaluate the apparent diffusion coefficient (ADC)-values. The results of the DWI as well as MRS were compared retrospectively with the findings of the histological examination. Sensitivity and specificity of ADC and metabolic ratio (MET)—both separately and in combination—for identification of tumor tissue was computed for variable discrimination thresholds to evaluate its receiver operator characteristic (ROC). An association between ADC, MET and Gleason score was tested by the non-parametric Spearman *ρ*-test. *Results.* The average ADC-value was 1.65 ± 0.32mm^2^/s × 10^−3^ in normal tissue and 0.96±0.24 mm^2^/s × 10^−3^ in tumor tissue (mean ± 1 SD). MET was 0.418 ± 0.431 in normal tissue and 2.010 ± 1.649 in tumor tissue. The area under the ROC curve was 0.966 (95%-confidence interval 0.941–0.991) and 0.943 (0.918–0.968) for DWI and MRS, respectively. There was a highly significant negative correlation between ADC-value and the Gleason score in the tumor-positive tissue probes (*n* = 62, *ρ* = −0.405, *P* = .001). MRS did not show a significant correlation with the Gleason score (*ρ* = 0.117, *P* = .366). By using both the DWI and MRS, the regression model provided sensitivity and specificity for detection of tumor of 91.9% and 98.3%, respectively. *Conclusion.* The results of our study showed that both DWI and MRS should be considered as an additional and complementary tool to the T2-weighted MRI for detecting prostate cancer.

## 1. Introduction

In Europe as well as in the United States prostate cancer is a very common and frequent cancer in males. According to the American Cancer Society, it is the third leading cause of cancer-related death in men. In 2007, 218,890 new cases are assumed to be diagnosed and about 27,050 persons estimated to die due to this disease [1]. In Europe the incidence of the prostate cancer is approximately 30 per 100 000 men and also the third frequent cause of death after lung and colorectal cancer [2]. 

Several diagnostic methods have been applied in recent years to detect the malignant changes within the prostate. The transrectal ultrasound (TRUS) of the prostate is the primarily used method worldwide, besides the laboratory modus operandi. It is used not only to gain the first impression of the organ, but also to guide prostate biopsies, if necessary [3]. Another commonly used method is MR imaging of the prostate using an endorectal coil and a pelvic phased-array coil, in which the malignant sites usually show a hypointense signal compared to the normal hyperintense peripheral zone [4]. In contrast to the TRUS, the patients have to undergo longer examinations, but the local staging as well as the assessment of the surrounding tissues and organs has shown a better sensitivity using the MRI than the aforementioned [5]. Recent studies also showed that the MR-guided biopsies of the prostate are possible and are of the same histopathologic quality as specimens obtained with a TRUS guided biopsies [6].

Over the past few years, however, other MRI techniques have been developed to improve the diagnostic accuracy. A number of studies have shown that the three-dimensional ^1^H-spectroscopy of the prostate can ameliorate the anatomical and morphological situation as well as the characterization of the prostate cancer [7]. 

Diffusion weighted MR Imaging (DWI) is a technique to evaluate the molecular diffusion based on the Brownian motion of the spins in biological tissues. DWI provides information on both the perfusion and the diffusion in any organ to characterize abnormal tissue changes within the sites. This method can be regarded as an additive method to T2-weighted MRI by developing image contrast through “apparent diffusivity.” Diffusion weighted MRI is showing potential for improving prostate cancer detection [8–10]. By adding the diffusion-weighted imaging to conventional T2-weighted MR imaging, an improvement of detection of prostate cancer was found [8], and diffusion-weighted imaging at 3.0 T has also showed reduced ADC values and increased fractional anisotropy in prostate cancer [11] as well.

These different methods were combined and compared in several studies as well, especially the MR spectroscopy and the DW imaging. It has been shown that there is a positive correlation between ADC values and the ratio for choline and creatine to citrate in men with elevated prostate-specific antigen (PSA) levels [12]. Another study showed that if an examined voxel contained ≥70% tumor, the combined usage of MR spectroscopy and DW imaging increased the specificity in detecting prostate cancer, while the sensitivity compared to MR spectroscopy or DW imaging alone retained [13]. Recently, Mazaheri et al. has reported a more precise study about the same issue, using the three-dimensional (3D) hydrogen 1 (^1^H) MR spectroscopy and the DW imaging. Also in this study, it could be shown that the combination of these two had a significant improvement in differentiation from malignant and benign tissue [14]. 

The aim of this study was to apply both (3D) hydrogen 1 (^1^H) MRS and DWI to the prostate and to determine the Choline-Citrate ratios and the ADC values of healthy tissue and prostate cancer and to compare retrospectively the results with histology, by means of the Gleason-Score, in patients with questionable prostate cancer. Herewith, we hope to assess the potentials with regard to the differentiation of cancer, and to determine the ADC values and the Choline-Citrate ratio of healthy tissue and prostate cancer in comparison to histology.

## 2. Materials and Methods

### 2.1. Study Population

In this study, 50 patients with clinical suspicion of prostate cancer underwent a combined endorectal-body-phased-array MRI at a 1.5 T MRI scanner. The mean age of the examined patients was 61.8 years, with the range of 44 to 78 years. The study protocol was approved by the local Ethics Committee, and informed consent was obtained from all patients. From all patients, blood samples were taken to ascertain the prostate specific antigen (PSA) levels. The prerequisite for the examination was that the patients would undergo a transrectal ultrasound (TRUS) and biopsy or prostatectomy thereafter. Patients with prior hormonal, surgical, or irradiation therapies as well as previous biopsies within 12 weeks prior to the examination date were excluded.

### 2.2. MRI Imaging Protocol

All examinations were performed on a 1.5 T scanner (Symphony; Siemens Medical Solutions, Erlangen, Germany) with a combination of an endorectal coil (MRInnervu; Medrad, Indianola, USA) and a body and spine panoramic array. No contrast medium was used. For the morphological evaluation of the prostate including the lymph node status of the pelvis, a T1-weighted spin echo (SE) sequence was used. To evaluate prostatic changes a T2-weighted fast spin echo (FSE) sequence in transversal, coronal, and sagittal orientation was performed (Table 1).

### 2.3. Diffusion-Weighted Imaging

Based on the T2w images a diffusion weighted (DW) spin echo-planar sequence was generated in transversal orientation to include the whole prostate using the following parameters also using the above-mentioned coil combination: TR 3100 ms; TE 88 ms; FOV 180 × 180 mm; matrix 128 × 128 mm; Slice thickness 4 mm; intersection gap 0 mm; voxel size 1.8 × 1.5 × 4 mm; *b*-factors 50, 400, 800 s/mm^2^; 20 slices. The duration of the examination was about 4 to 6 minutes. For DW imaging the above-mentioned coil combination was used.

The ADC is given by the following equation:

(1) S(I)=S(0)e−(bi·ADC),

where *S*(*I*) was the signal intensity measured on the *i*th *b-*factor image, and *b*_1_ was the corresponding *b*-factor. *S*_0_ estimates the signal intensity for a *b*-factor of 0 s/mm^2^, that is, without the noise induced by the MR measurement [15]. A starting *b*-value of 50 s/mm² was used to suppress vascular signal in the initial T2 weighted EPI image. The diffusion weighting was performed with a trace weighted sequence type (3 orthogonal directions).

According to this equation, ADC-maps were generated using the software attached to the scanner on the basis of a voxelwise calculation and were interpolated to a 256 × 256 mm matrix.

### 2.4. 3D-^1^H MR Spectroscopic Imaging

The spectroscopic imaging was performed with the spectroscopic software provided by the MR scanner (Symphony; Siemens Medical Solutions, Erlangen, Germany), using only the endorectal coil. This software acquires data with the point-resolved spatially localized spectroscopy. By using spectral-spatial pulses, choline, creatine, and citrate were excited within the box. Water and lipids were suppressed with a shim around the spectral box. The box was placed on the transverse T2 weighted images, corresponding to the images made beforehand. The magnetic field homogeneities were automatically optimized by shimming algorithms provided by the manufacturer. 

The following parameters were acquired for the MR spectroscopy: TR 700 ms; TE 120 ms; Flip angle 90°; number of signal acquired = 1; spectral width = 1300 Hz; number of points = 512; FOV 80 × 80 × 80 mm^3^, and phase-encoding steps = 16 × 8 × 8. The voxel volume was 6.7 × 6.7 × 6.7 mm^3^; SNR 100 csi-ce. The duration of the whole MR spectroscopy was about 11.46 minutes. 

The evaluation of the spectral data was made by utilizing the manufacturer's postprocessing software package. The postprocessing included zero filling of the raw data in the superior-inferior direction with a four-dimensional Fourier transformation to yield a voxel volume of 300 mm^3^, spectral apodization with a 2 Hz Lorentzian function, base line correction, peak registration, and an alignment of 3D ^1^H MR spectroscopic images to the transverse T2 weighted images. The diameter of integration was 0.3 ppm and was adjusted for each voxel, just to reach the optimal broadening of each spectral peak. Metabolic ratio maps of choline, creatine, and citrate were generated: (choline + creatine)/citrate = MET (metabolic ratio).

The whole time duration of the MRI examination (incl. MRI, DWI and MRS) including time for patient placement, coil placement, and localization of the prostate was approximately 40 minutes. The image acquisition time for the T1 SE, and the T2 FSE was 24 minutes and that for the DWI were 5 minutes. The MRS lasted approximately 12 minutes.

### 2.5. MR Image Analyses

The morphological and possibly pathological sites of the peripheral zone of the prostate were retrospectively assessed with the T2 weighted images by dividing the prostate into sextants, that is, the apex, the mid-portion, and the base, each right and left side. Then, the grey value of the pixel corresponded to the ADC value [mm^2^/s × 10^−3^] since a pixel-to-pixel ADC map was automatically calculated for each slice. The value itself was calculated with the equation mentioned above. The regions of interest (ROI) were then manually drawn in each sextant of the prostate guided by the T2 weighted images. The mean ROI size was 0.8 mm^2^ (SD ± 0.56). Additionally, metabolic ratio maps of choline, creatine, and citrate were generated with the manufacturer's software package for each voxel, especially of suspect areas with the MR spectroscopy. Voxels were classified as suspicious if the MET was >0.86 [16].

### 2.6. Biopsy/Histopathologic Analyses

All patients underwent TRUS guided biopsies (in sextants: right and left sites of the apical, the mid-partial and the basis of the prostate). All biopsies were performed by urologists, and the biopsy cores were labelled to specify the location of the biopsy. Histopathologic analyses were made by the Institute of Pathology for all biopsies of the prostate and the Gleason scores were evaluated.

### 2.7. Statistical Analyses

The prostate was divided into 6 regions, that is, sextants: right/left apex, right/left midsection, and right/left base. Tissue probes from each site were classified as “normal tissue” or “tumor tissue” according to histopathology. Tumor tissue was further categorized according to the Gleason score.

Univariate analysis of variance with ADC-values as independent variable and tissue type (normal, tumor) and as intersubject factors was used to compare the ADC value between normal tissue and tumor and between different sextants.

Sensitivity and specificity of ADC-values for identification of tumor tissue were computed for variable discrimination thresholds to evaluate its receiver operator characteristic (ROC). The histologically determined tissue type served as gold standard. The area under the ROC curve was computed as an overall performance measure. An association between the ADC value and Gleason score was tested by the nonparametric Spearman *ρ*-test. The analysis was restricted to the tumor-positive probes.

The statistical analyses were repeated with MET instead of ADC-value as independent variable.

An effect was considered statistically significant if the significance level *α* = 0.05 was reached. All statistic computations were performed using SPSS 15.0.1 for MS Windows.

In order to test whether the combination of DWI and MRS might improve the accuracy for detection of tumor tissue compared to both DWI and MRS alone, stepwise binary logistic regression was used with histopathology (normal tissue, tumor) as dependent variable and ADC values and MET as possible regressors (*P* for inclusion .05, *P* for exclusion .10).

## 3. Results

The mean value of the prostate specific antigen (PSA) taken from blood samples was 7.19 *μ*g/L (SD ± 5.2), where the mean PSA level in patients with prostate cancer was 10.41 *μ*g/L (SD ± 5.1) and those in healthy ones 4.1 *μ*g/L (SD ± 4.2). 

**Figure 1:**
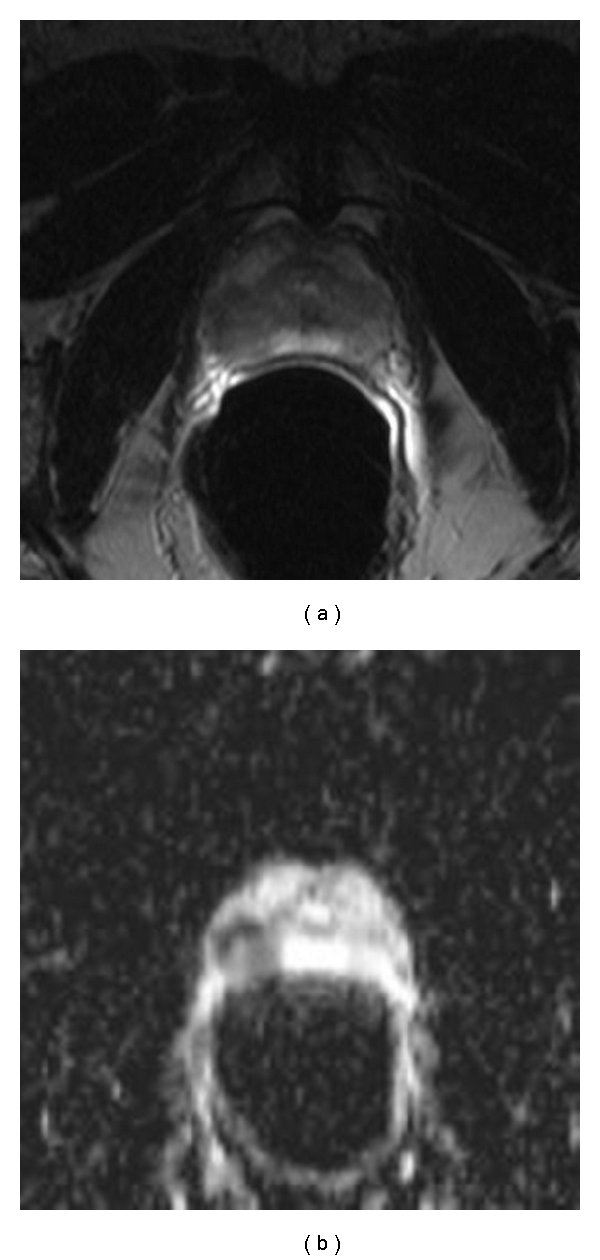
Patient (45 y.o.) with prostate cancer (2.6 cm): PSA-level 9.7 *μ*g/L (free PSA-level 14.56 *μ*g/L); Gleason-Score 4 + 5 = 9. In the T2w image (a) the prostate cancer is demonstrated in the right peripheral zone. In the correspondent ADC-map (b) the prostate cancer is clearly shown as a hypointense area. The left peripheral zone looks hyperintense on the T2w image, but the ADC-map reveals the remaining healthy prostate tissue.

All MR examinations were performed successfully, and although some images had susceptibility artifacts due to the endorectal coil, all images could be used for analyses. The image acquisition time for the T1 SE, and the T2 FSE was 24 minutes and that for the DWI were 5 minutes. The MRS lasted approximately 11 minutes. As demonstrated in Figure 1 the ADC maps show hyperintense in benign and hypointense in malignant tissue in the peripheral zone. 

Histopathology identified tumor tissue in 21 of the 50 patients (42%). All 6 sextants were infiltrated by the tumor in 3 of these patients, 4 sextants in 3 patients, 3 sextants in 5 patients, 2 sextants in 7 patients, and in 3 patients tumor tissue was detected in only 1 sextant. Thus, in total 62 of the 300 tissue probes were tumor positive according to histopathology (20.7%). 

The rate of tumor-positive tissue probes ranged between 14% (left apex) and 28% (right midsection). However, this variation was not significant statistically (Pearson's “portmanteau” *χ* ^2^ test: *χ* ^2^ = 4.066, *df* = 5, *P* = .540).

The Gleason score of the tumor-positive tissue probes was 5 in 3 sextants (4.8%), 6 in 16 sextants (25.8%), 7 in 22 sextants (35.5%), 8 in 6 sextants (9.7%), 9 in 11 sextants (17.7%), and 10 in 4 sextants (6.5%).

**Figure 2:**
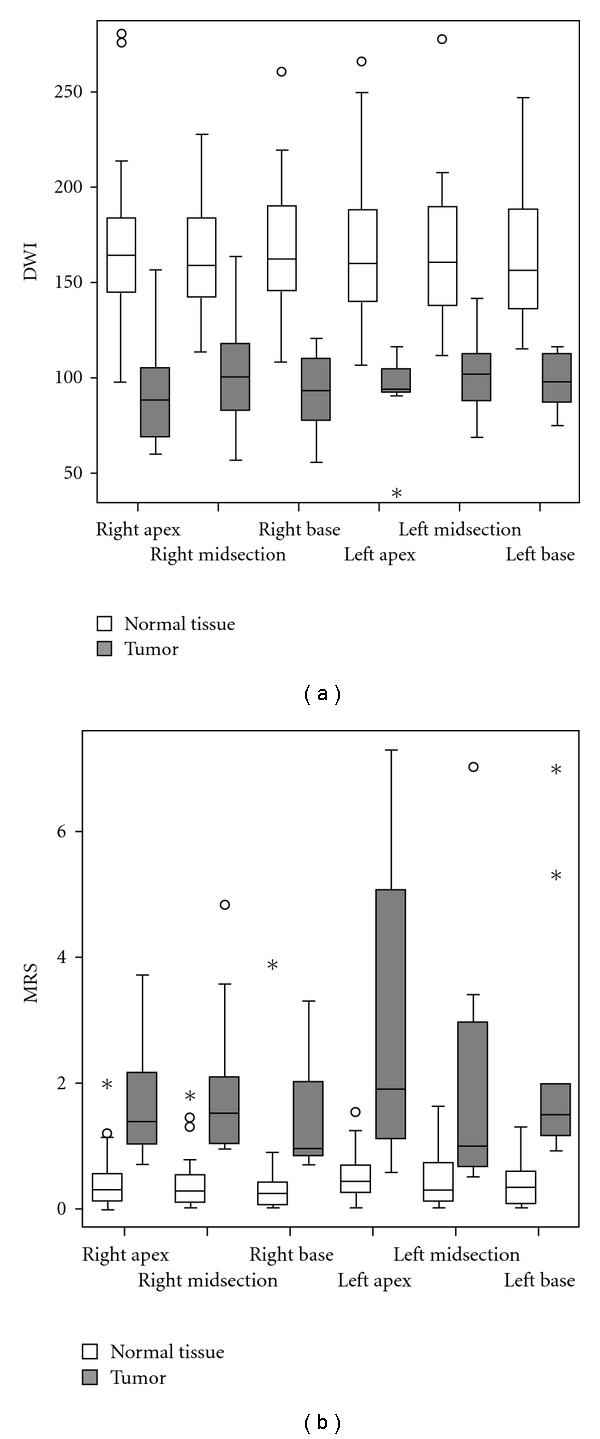
Box-and-whisker plot of DWI (a) and MRS (b) as a function of tissue type (normal tissue, tumor) and region of interest (ROI). Outliers (1.5–3 box lengths) are indicated by a circle; extreme values (>3 box lengths) are indicated by an asterix.

Univariate analysis of variance revealed a highly significant difference of ADC-value between normal tissue and tumor tissue (*F* = 224.5, *df* = 1, *P* = .000), but no difference between the sextants (*F* = 0.138, *df* = 5, *P* = .983) (Figure 2). There was also no significant interaction effect of tissue type and sextants on ADC values (*F* = 0.356, *df* = 5, *P* = .878). Averaged overall sextants, ADC-value was 1.65 ± 0.32 in normal tissue and 0.96 ± 0.23 in tumor tissue (mean ± 1 standard deviation).

MET also showed a highly significant difference between normal tissue and tumor tissue (*F* = 198.4, *df* = 1, *P* = .000) (Figure 2). The analysis of variance suggested a significant effect of the sextants (*F* = 4.5, *df* = 5, *P* = .001) as well as a significant interaction effect of tissue type and sextants on MRS (*F* = 3.4, *df* = 5, *P* = .006). However, post hoc comparison of MET between any pair of sextant did not reveal any significant effect, neither in normal tissue nor in tumor. Therefore, the sextants were not taken into account in the further analyses. MET was 0.418 ± 0.431 in normal tissue and 2.010 ± 1.649 in tumor tissue.

## 4. Sensitivity and Specificity of DWI and MRS Separately and Combined

Sensitivity and specificity of DWI and MRS were evaluated separately using ROC analysis; a combined evaluation of both methods was performed using a stepwise binary logistic regression.

**Figure 3:**
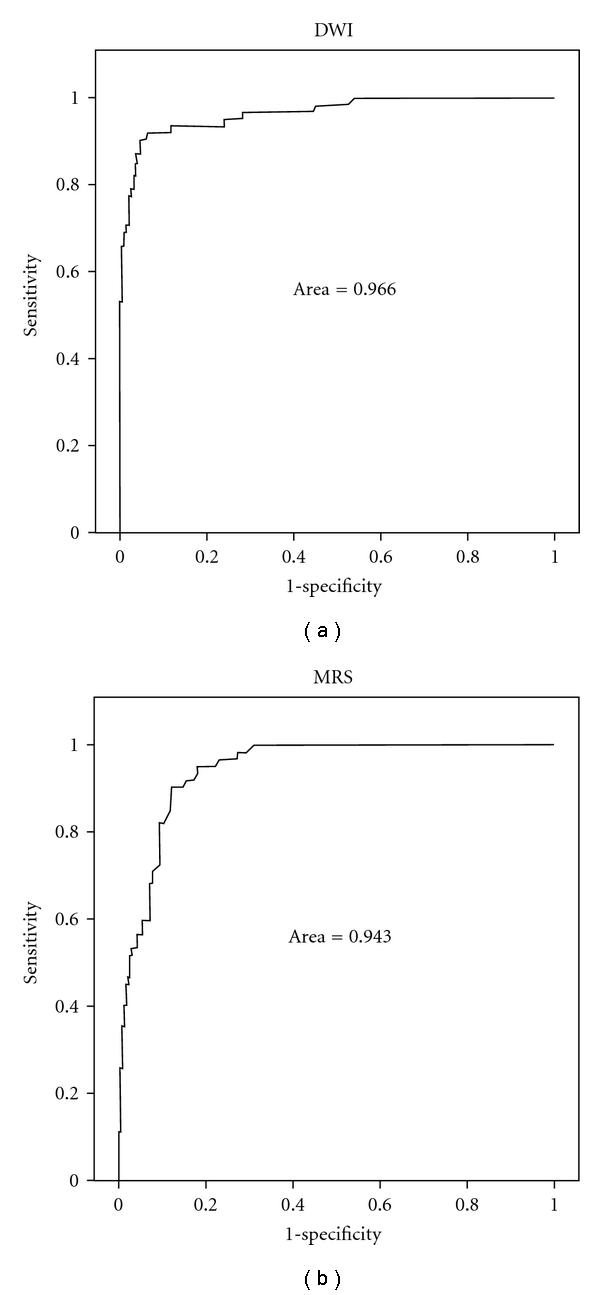
Receiver operator characteristic (ROC) curve of DWI (a) and MRS (b) for differentiating tumor tissue from normal tissue. The analysis included all 300 tissue probes irrespective of the ROI.

The ROC curves of DWI and MRS for identification of tumor tissue irrespective of the sextants are given in Figure 3. The area under the ROC curve was 0.966 (95%-confidence interval 0.941–0.991) and 0.943 (0.918–0.968) for DWI and MRS, respectively. For DWI a sensitivity of 0.92 and a specificity of 0.93 were provided using discrimination threshold of 1.208 mm^2^/s × 10^−3^. With a threshold providing the same sensitivity, that is, 0.92, MRS provided a specificity of 0.85. With a threshold providing the same specificity, that is, 0.93, MRS provided a sensitivity of 0.68. 

**Figure 4:**
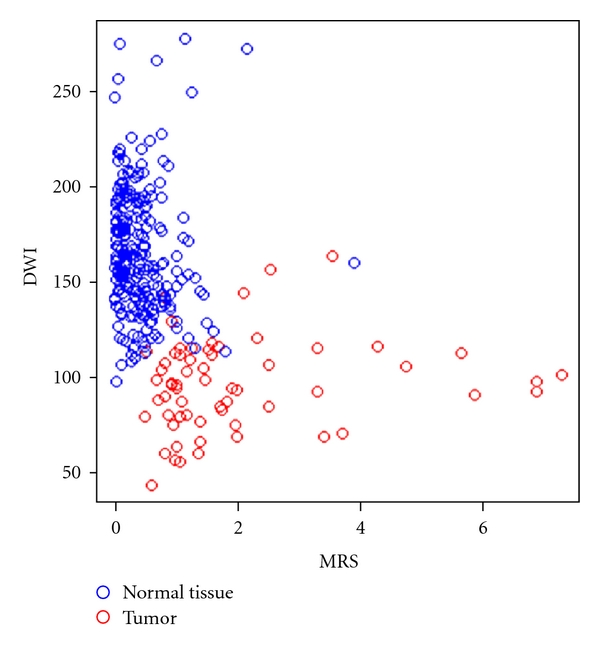
Scatter plot of DWI versus MRS in normal tissue probes and tumor-positive tissue probes.

Stepwise binary logistic regression included both DWI (*P* < .001) and MRS (*P* < .001) for the differentiation between normal tissue and tumor is demonstrated in Figure 4. The regression model classified 291 of the 300 probes correctly (97.0%). Only 4 of 238 normal tissue probes were misclassified as tumor, and 5 of 62 tumor probes were misclassified as normal. Thus, the regression model provided a sensitivity and specificity for detection of tumor of 91.9 and 98.3, respectively. 

**Figure 5:**
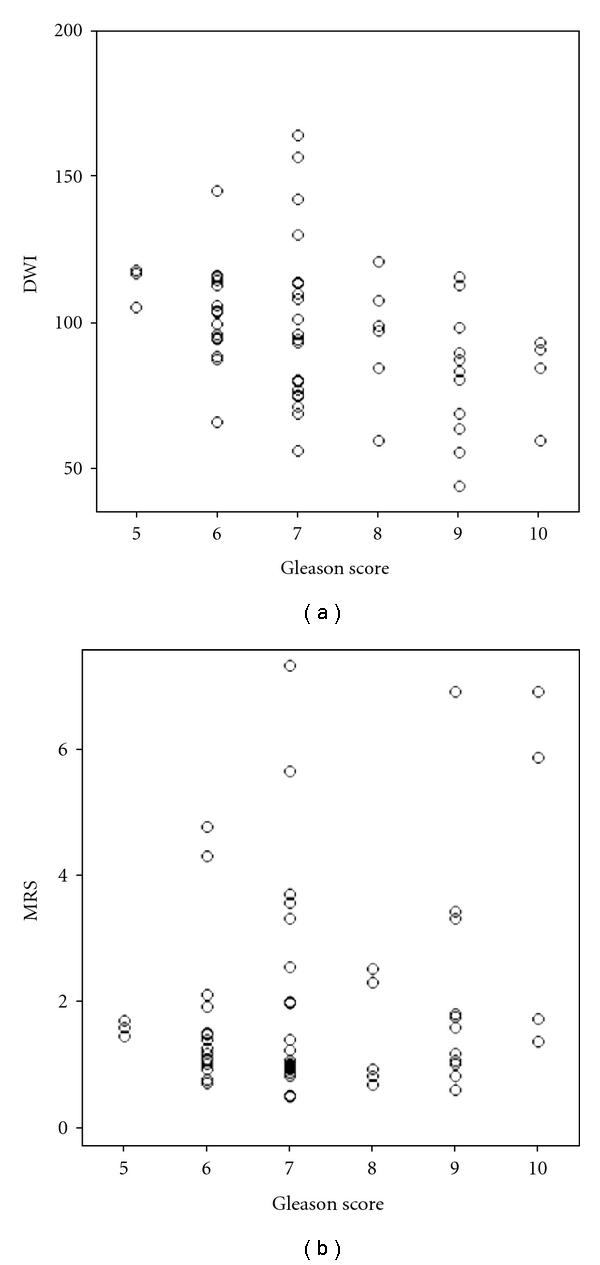
Scatter plot of DWI (a) and MRS (b) versus Gleason score in tumor-positive tissue probes.

There was a highly significant negative correlation between DWI and the Gleason score in the tumor-positive tissue probes (*n* = 62, *ρ* = −0.405, *P* = .001) (Figure 5). In contrast, MRS did not show a significant correlation with the Gleason score (*ρ* = 0.117, *P* = .366).

## 5. Discussion

However, the fact that the MRI of the prostate might be more advantageous than the transrectal ultrasound for staging the cancer has also been discussed in the past (especially for T2 and T3 tumours) [17, 18]. The T2 weighted MRI images of the prostate has been applied more often to improve the validity of the staging in prostate cancer [19]. The prostate is one of the few organs in humans which can be examined by MRI without any contrast media. Benign tissues in the peripheral zone show hyperintense signals in T2 weighted imaging, whereas malignant changes show hypointense signals, of which the reason could be the cellular density as well as the malfunction of the gland when the malignant change had occurred. The cause of the decrease in diffusion in malignant tissue has a histopathologic origin. Some attributes are: hypercellularity, enlargement of the nuclei, hyperchromatism, and angulation of the nuclear contour, which lead to a reduction of diffusional displacement of water molecules (Anderson JR. Muir's textbook of pathology. London, England: Edward Arnold 1985). Commonly, the prostate produces 20 to 30% of the ejaculate secretions. In patients with known prostatic cancer, the amount of the ejaculate can be less than in healthy patients. However, this is difficult to determine since the secretions vary from 0.5 to 13 mL. 

Diffusion weighted MR imaging has been clinically applied in several organs. Not only is it used to show the affected tissue after a stroke, it is also to differentiate brain tumours [20, 21] or also vertebral metastases in, for example, prostate cancer [22]. In diffusion-weighted MRI (DWI) the image contrast is determined by the random microscopic motion of water protons, that is, the Brownian motion. The diffusion can be measured in vivo by using the MRI because of its sensitivity to motion. This sensitivity to motion can be increased by the addition of strong magnetic field gradient pulses to the pulse sequence [23]. Shimofusa et al. applied the DWI of the prostate with parallel imaging and with a high *b*-value (*b* = 1000) for the first time [8]. In this study they have not used an endorectal coil, but the sensitivity as well as specificity was higher than in other former studies with endorectal coil [24–27] or a dynamic study [28]. Since then, several prostatic MR imaging modules were performed to increase the detectability of cancerous tissue. The results of these studies demonstrate that the ADC value may provide information about the malignant changes in the prostate [29–31]. 

MR spectroscopy is a relatively new method in diagnosing prostate cancer and has been a part of clinical routine since 1980s. Since then, MR spectroscopy has been effective in improving the accuracy of MR imaging in prostate cancer localization and staging [32–34]. In the healthy prostate, malign and benign tissues can be differentiated by the MR spectroscopy on the basis of the metabolic ratio of choline, creatine, and citrate. The ratio is calculated by the equation MET = (choline + creatine)/citrate. The MET is increased in malign tissue whereas a lower MET can be found in benign tissue [35, 36].

In this present study, 50 patients with suspected cancer of the prostate were examined with the DW MRI and the MR spectroscopy, and then compared retrospectively with pathohistological results, especially with the Gleason score. 

Two recent studies compared and analyzed the combined usage of diffusion-weighted MRI and ^1^H MR spectroscopy. The one study examined 42 patients with prostate cancer using a 2D chemical shift imaging and isotropic apparent diffusion coefficient (ADC) maps [13]. In this study the regions of interest were drawn around the whole gland, central gland, and the peripheral zone tumor. The mean ADC value of the normal tissue in this study was 1.51 mm^2^/s × 10^−3^ [SD: ±0.27].

If the tumor was greater than 30% of the whole voxel the mean ADC value was 1.19 mm^2^/s × 10^−3^ [SD: ±0.24], and if the tumor was greater than 70% of the whole voxel, the value was 1.03 mm^2^/s × 10^−3^ [SD: ±0.18]. The mean MET in normal gland was 0.065 ± 0.052, whereas the value was much higher in malignant tissues; 0.814 ± 2.202 in tumor ≥30% of the voxel and 0.917 ± 1.276 in tumor ≥70%, respectively. The MET was significantly higher (*P* < .001) and the ADC values were significantly (*P* < .006) lower in tumor-containing voxel. The area under the ROC curves using both the ADC and MET was 0.81, similar to only MET (0.79), whereas ADC alone showed an area of 0.66 and was inferior. An interesting point, however, is the significant improvement in specificity for the combination of ADC and MET, when voxels containing 70% or tumor were considered positive and cutoffs to achieve a 90% or greater sensitivity were chosen [13].

The other study performed a retrospective measurement of ADC and MET in 38 patients with prostatic cancer. The mean ADC value and MET for malignant tissue were 1.39 mm^2^/s × 10^−3^ [SD: ±0.23] and 0.92 ± 0.32, respectively. For benign tissue, the values were 1.69 mm^2^/s × 10^−3^ [SD: ±0.24] and 0.73 ± 0.18 (*P* < .001 for both). In this study, areas under the receiver operating characteristic curves (AUCs) were performed to evaluate the accuracy. Obviously, the combination of ADC and MET performed significantly better (AUC = 0.85; *P* = .005) than ADC or MET alone (AUC = 0.81 and AUC = 0.09, resp.) [14].

Analogue to these prior studies, our results of the ADC values were significantly lower (*P* < .001) and the results of the mean MET were significantly higher (*P* < .001) for malignant prostatic tissues than for benign tissues. In our study, the average ADC value in benign tissue was 1.65 mm^2^/s × 10^−3^ [standard deviation (SD): ±0.32] and that in malign tissues 0.96 mm^2^/s × 10^−3^ [SD: ±0.24]. The significantly (*P* < .001) lower ADC values in malign tissues compared with the benign signify some promising results in detecting the cancer. These results were similar to other prior studies, although different *b*-values (0 and 1000 s/mm^2^ and 0, 300, 600 s/mm^2^) [30, 37] were used in our study (i.e., 50, 400, 800 s/mm^2^). The cutoff value of the mean ADC value between cancerous and noncancerous tissue in the present was at approximately 1.2 mm^2^/s × 10^−3^, which can be seen on the ROC-Analyses (Figure 3). Compared to the aforementioned studies our results of the ADC value for malignant tissue were remarkably lower. The MET of malignant tissues showed an average value of 2.010 ± 1.649 in our study, and is significantly higher than in healthy tissues (0.418 ± 0.431). This result can also be compared with previous studies. 

In this present study, a relatively larger number of patients were examined than other studies. The combination of DWI and MRS performed significantly better in detecting cancer in the periphery zone of the prostate than MRS alone [14] in one study. In our study with 50 patients, the combination of DWI and MRS seems also to have a better accuracy in detecting cancerous tissue. The regression model classified 291 of the 300 probes correctly (97.0%). Only 4 of 238 normal tissue probes were misclassified as tumor, and 5 of 62 tumor probes were misclassified as normal. Thus, the regression model provided sensitivity and specificity for detection of tumor of 91.9 and 98.3, respectively (Figure 5).

If the results were correlated with the Gleason-score, there was a highly significant negative correlation between DWI and the Gleason score in the tumor-positive tissue probes (*n* = 62, *ρ* = −0.405, *P* = .001) (Figure 4). However, MRS did not show a significant correlation with the Gleason score (*ρ* = 0.117, *P* = .366). One explanation could be that in the present study the MET values were extracted using a very short TR sequence (700 ms) which keeps the scan time tolerable but results in spectra that are fairly heavily T1-weighted. Perhaps that is why the other groups [38] found a weak correlation between Gleason and MET.

There are some problems and limitations in our study like in other MR studies of the prostate. First limitations are based on MR technique itself. Common artefacts for the DWI are white pixel noise, low SNR (signal-to-noise ratio), and susceptibility artefacts. In MR spectroscopy common artefacts are lipid contamination and susceptibility artefacts. These artefacts could be reduced by new methods in the future. Second limitation is the discrepancy in voxel sizes between the ADC map and the 3D ^1^H MR spectroscopy. In the future new spectroscopic techniques with increased spatial resolution without increasing the examination duration [39] could be used to overcome this problem. The present study was a retrospective analysis of the prostate cancer. The malignant sites were known when analyzing the ADC map and the MET. Chronic changes, such as chronic prostatitis or atrophy of the gland itself, usually show similar changes in MRI [4, 27] to prostatic cancer. Prospective studies are another research task for succeeding studies with the DWI and MRS, especially in patients with several negative biopsies. The last limitation of this study was the usage of sextant biopsy, since biopsies can be easily false negative so that the tumor localization might not be very exact. These methods can then be combined with the dynamic contrast-enhanced magnetic resonance imaging, since first studies have shown that it may be an accurate technique for detecting and quantifying intracapsular transition or peripheral zone tumor foci greater than 0.2 cc [40].

## 6. Conclusion

The results of our study showed that both diffusion-weighted Imaging and MR spectroscopy should be considered as an additional and complementary tool to the T2 weighted MRI not only for detecting prostate cancer, but also for guiding more specific biopsies without taking several samples from the organ. The combination of these methods can improve the specificity and may prevent uncomfortable as well as painful biopsies for patients. The DWI is furthermore advantageous over the present examination methods, such as the MR spectroscopy, regarding the considerably shorter examination time (approximatly 5 minutes in case of this study) and the correlation with the Gleason score.

## Figures and Tables

**Table 1:** 

Sequence	TR [ms]	TE [ms]	Slice Thickness [mm]	FoV [mm]	Matrix
T1 SE	765	14	5	350	215 × 215
T2 FSE transverse	3400	98	3	180	205 × 256
T2 FSE sagittal	3000	98	3	200	205 × 256
T2 FSE coronal	3000	98	3	200	205 × 256
